# Efficacy analysis of virtual reality-based training for activities of daily living and functional task training in stroke patients: A single-subject study

**DOI:** 10.1097/MD.0000000000033573

**Published:** 2023-04-21

**Authors:** Lan-Ju Lee, Seong-Youl Choi, Hye-Sun Lee, Sang-Woo Han

**Affiliations:** a Department of Occupational Therapy, Gwangju Heemang Hospital, Haseo-ro, Buk-gu, Gwangju, Korea; b Department of Occupational Therapy, Kangwon National University, Dogye-eup, Samcheok-si, Gangwon-do, Korea; c Department of Occupational Therapy, Kwangju Women’s University, Yeodae-gil, Gwangsan-gu, Gwangju, Korea.

**Keywords:** cognitive function, performance of daily activities, stroke, upper extremities function, virtual reality

## Abstract

Virtual reality (VR)-based training for functions such as cognition, upper extremities, balancing, and activities of daily living (ADL) has been used on stroke patients, and its efficacy has been reported. However, no comparison has been made between the efficacy of VR-based training for daily activities that exactly reproduces ADL and functional training. Therefore, this study sought to analyze the difference in independency enhancement of VR-based training for daily activities compared to cognitive and motor functional training.

**Patient concerns and diagnosis:**

This study was conducted on 4 patients who have been diagnosed with stroke and are currently receiving rehabilitation therapy in G hospital located in the city of Gwangju, using A-B-A’-B’ design from single-subject experimental designs.

**Interventions:**

Intervention was performed in 2 ways: application of VR-based training for daily activities after the application of cognitive and motor function training; and application of cognitive and motor function training after the application of VR-based training for daily activities. The Assessment of Motor and Process Skills, Computer Cognitive Screening Assessment System, Box and Block Test, and Grip and Pinch Strength Test were used to measure the changes in the performance of daily activities, cognitive function, and upper extremities function.

**Outcomes:**

The results confirmed that the performance of daily activities, cognitive function, and upper extremities function was improved after the application of VR-based intervention. In addition, the efficacy of independency enhancement was maximized by the early approach of training for daily activities at the time of VR-based intervention in stroke patients.

**Conclusions:**

VR-based intervention of training for daily activities and functional training can be considered to benefit the improvement of the performance of daily activities, cognitive function, and upper extremities function in stroke patients. In addition, although functional training was also effective in enhancing independency and functional improvement in stroke patients, an early approach to training for ADL based on tasks with objectives was deemed to be more effective.

## 1. Introduction

Stroke is a disease that develops following vascular damage of the brain, and accompanies impairment of motor function, consciousness level, cognition, perception, and language.^[1]^ Eighty-five percent of stroke patients experience hemiplegia, with 69% of these having motor function impairment of the upper extremities^[2]^ and will have a permanent disability if not treated adequately.^[3]^ In addition, cognitive impairment due to stroke leads to a decline in attention, memory, and spatial perception.^[4]^ This presents a problem with processing significant stimulations provided by the environment and interferes with activity performances worthy of oneself.^[5]^

These complex impairments result in difficulty performing daily activities such as maintaining personal hygiene, eating, and dressing. They also affect the comprehensive coordination of daily activities, leading to a dependent life.^[6]^ In addition, difficulties in independent living cause changes in autonomy and personal role, which in turn cause long-term stress and psychological withdrawal, leading to decreased quality of life and social issues.^[7]^

Occupational therapy helps stroke patients to improve independence through adequate and meaningful activities and therapeutic application with goals and also helps them to adapt actively to their new environment.^[8]^ The intervention focused on the improvement of individual functions such as upper extremities function, cognitive function, and balancing is important, but supporting their adaptation to a new environment and improvement of independent performances through occupational performance-based intervention is critical for the promotion of independence, which is the ultimate goal of occupational therapy.^[9]^ Education and training in occupational performance play a key role in the field of occupational therapy, and it is significant that the patient is provided directly with an opportunity to receive an intervention.^[10]^ Strategies obtained from rehabilitation therapy can thus be transferred or applied to other tasks or circumstances, and generalization can be set as an objective.^[11]^

For these reasons, it can be ideal to minimize errors following the patient’s application of strategies achieved through rehabilitation therapy to real-life environments or to popularize the intervention for generalization of searching efficient adaptation strategies.^[12,13]^ In order to popularize the intervention for generalization of performance techniques for a patient in real life environment, efforts to provide occupational therapy services have been made through home visit rehabilitation programs in Korea recently.^[14,15]^ This helps patients who have difficulties in independent living due to functional impairment by providing professional and systemic rehabilitation therapy, therefore increasing their quality of life and allowing them to participate in the local community.^[16]^

It will be beneficial if such intervention for generalization can be applied to all patients, but home visit occupational therapy is not actively performed compared to nursing or physical therapy services, although the demand is high.^[17]^ Therefore, intervention for generalization and occupational performance during the recovery period before returning to real life environment is necessary for the patient to maintain actual daily living such as daily activities, adaptation to the home environment, adaptation to the community, and hobbies in a familiar environment.^[18]^

Virtual reality (VR)-based intervention has the benefit of overcoming limitations concerning time and space, which had been the issue with previous interventions and training, and is therefore being utilized in various fields.^[19]^ Recently, VR-based intervention for tasks of daily living is also being utilized, and this is being suggested as an alternative for stimulating generalization in patients who are in the recovery stage.^[20]^

A VR-based intervention program is a simulation technique that reproduces the reality in which the user lives and allows a similar experience, using computers and specialized equipment.^[21]^ This interests and motivates the user, while providing an environment for training that is similar to reality. In addition, education can be more effective because the difficulty level of task performance can be adjusted, and immediate feedback for the achievement level of the task performance is available.^[22]^ Opportunities for various occupational activities can be provided, and concentration training, repetitive training, and task-centered training can be performed, positively influencing stroke patients.^[23]^

Therefore, because a VR-based intervention program provides interactive, repetitive, and continuous skill training in a virtual environment, it is deemed adequate as a method of intervention during the recovery period.^[24]^ As previously mentioned, for the enhancement of independency level in patients whose lifestyles have been changed, the improvement and recovery of various functions are important, but the application of these functions to real life is more important than the functions themselves.^[25]^ In a VR-based intervention, occupational task performance in real life can be applied and utilized as a strategy to maximize generalization. Therefore, functional training that focuses on task application of cognitive function and motor function, or daily activities training that is based on tasks occurring in real life, are different from VR-based intervention in terms of efficacy and objective.^[26,27]^

Recently, VR-based intervention is being introduced as a method of therapeutic approach for the functional improvement of patients in the field of rehabilitation therapy.^[28]^ VR-based intervention and monitor-based VR game intervention improve the cognitive function and performance of daily activities in stroke patients.^[29]^ As a result of analyzing the difference in the improvement of upper extremities function between the group to which the motion-type VR-based intervention program was applied and the group to which general therapy was applied, the motion-type VR-based intervention program showed more favorable results in the improvement of upper extremities function in stroke.^[30]^ When the VR-based intervention program was applied to stroke patients, significantly high improvement in balancing and performance of daily activities was observed.^[31]^ The group to which the VR-based intervention program was applied showed greater improvement in cognitive function, attention, memory, time-spatial function, and performance, while the group to which general intervention was applied showed improvement only in memory and social participation.^[32]^ The VR-based intervention program was effective in improving the upper extremities function when applied to stroke patients.^[33]^ This signifies that VR-based intervention is related to clinically important improvements.

Many studies uniformly describe that VR-based functional training resulted in significant improvement of individual functions such as cognition, upper extremities and balancing, and performance of daily activities, leading to improvement of independence. However, no studies have been reported confirming the respective efficacy of functional training and VR-based training that utilizes tasks that reproduce the performance of daily activities in real life, or studies that compare the efficacy of the 2 types of training.^[20,30,33–36]^

Therefore, this study will utilize the VR-based intervention program “MOTOCOG,” divide the subjects into 1 group to which training for daily activities is applied first, and another group to which training for cognitive and motor function is applied first, and observe the changes in the performance of daily activities and changes in function, thereby proving the following hypothesis.

First, the efficacy of VR-based intervention for improvement in the performance of daily activities, cognitive function, and upper extremities function will be confirmed.

Second, VR-based training of daily activities and training of cognitive and motor function for the improvement of independence of stroke patients will be compared, and the differences will be analyzed.

## 2. Methods

### 2.1. Study design and process

**Figure 1.**
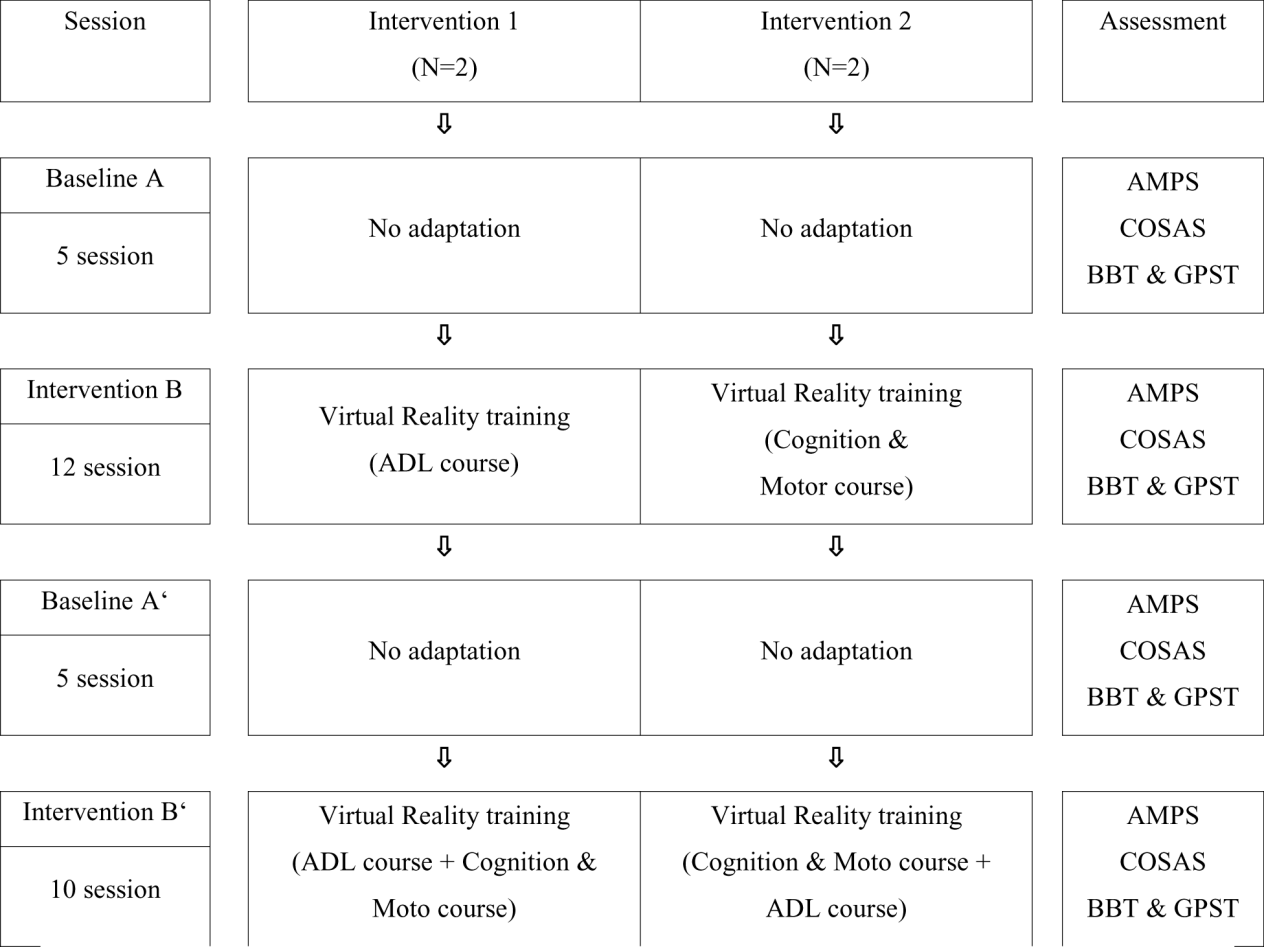
Study procedure.

This was a single-subject study that was constructed to A-B-A’-B’. The study period was from July 5 to November 16, 2021, and was conducted 5 times a week. The intervention was performed for 30 minutes per session. At each session, changes in the performance level of daily activities, cognitive function, and upper extremities function of subjects were observed. The overall procedure of the study is presented in Figure 1.

#### 2.1.1. Baseline A.

Baseline period A consists of 5 sessions, no VR-based intervention program was provided, and the performance level of daily activities, cognitive function, and upper extremities function of subjects were repeatedly measured.

#### 2.1.2. Intervention B.

Intervention period B consists of 12 sessions, and after the application of the VR-based intervention program, the repetitive measurement procedure used during the baseline period was adopted to measure the changes in the performance level of daily activities, cognitive function, and upper extremities function. For the VR-based intervention program, the subjects were divided into Group 1 (subjects 1, 2) and Group 2 (subjects 3, 4), and only the training for daily activities was applied to Group 1 while training for cognitive function and motor function was applied to Group 2.

#### 2.1.3. Second baseline A’.

Baseline period A’ consists of 5 sessions, no VR-based intervention program was provided, and the performance level of daily activities, cognitive function, and upper extremities function of subjects were repeatedly measured as in Baseline A.

#### 2.1.4. Second intervention B’.

Intervention period B’ consists of 10 sessions, and after the application of the VR-based intervention program, the repetitive measurement procedure used during the baseline period was adopted to measure the changes in the performance level of daily activities, cognitive function, and upper extremities function. For the VR-based intervention program, training for cognitive function and motor function was added to Group 1 of Intervention B, and training for daily activities was added to Group 2.

### 2.2. Participants

This study was conducted on 4 patients who have been diagnosed with stroke and are currently receiving rehabilitation therapy in G Hospital located in the city of Gwangju. The informed consent has been waivered by the Research Ethics Review Committee of the Kwangju Women’s University with the reason for the experiment place. However, this study was conducted in compliance with the research ethics of the Helsinki Declaration with the approval of the hospital to which the patient belongs. Written and oral information concerning the study was provided to all subjects and their guardians. All subjects voluntarily gave written consent to participate in the study. All participants understood that they could withdraw their participation for some reason during the study. The number of participants was determined in consideration of the single case study design. The 4 participants were randomly classified into intervention 1 and 2 application groups using the Excel program. The inclusion criteria for study subjects are as follows.

- Patients who were diagnosed with stroke by a specialist in rehabilitation medicine
- Patients who have no neurologic disease other than stroke
- Patients who were diagnosed with stroke >6 months prior to the study

All subjects were diagnosed with stroke by specialists >6 months prior to the study. Subject 1 had a left intracerebral hemorrhage, Subject 2 had a right cerebral infarct, Subject 3 had a right intracerebral hemorrhage, and Subject 4 had an unspecified intracerebral hemorrhage (Table 1). Unfortunately, the subjects who voluntarily participated in the study were all men. In addition, there was a difference in the age of the subjects, with 1 elderly, 2 middle-aged, and 1 young person. All subjects were receiving identical rehabilitation therapy including occupational therapy and exercise therapy twice a day.

**Table 1:** General characteristics of participants.

Participant		Gender	Age (yr)	Onset (mo)	Diagnosis	Paretic side	Dominant hand (pre/post)
Intervention 1	1	Male	48	23	Rt. hemiplegia d/t S-ICH on Lt. thalamus c IVH	Right	Right/Left
	2	Male	69	16	Lt. hemiplegia d/t Rt. MCA infarction	Left	Right/Right
Intervention 2	3	Male	42	26	Lt. hemiplegia d/t S-ICH on Rt. BG	Left	Right/Right
	4	Male	26	21	Lt. hemiplegia d/t Intracerebral hemorrhage, unspecified	Left	Right/Right

BG = basal ganglia, IVH = intraventricular hemorrhage, MCA = middle cerebral artery, S-ICH = spontaneous intracerebral hemorrhage.

### 2.3. Intervention

#### 2.3.1. VR-based intervention program (MOTOCOG).

MOTOCOG (Cybermedic, Korea) was the VR-based intervention program used in this study. This VR-based intervention program was developed to improve the performance level of daily activities, cognitive function, and motor function.^[37]^ It consists of training for daily activities, training for cognitive function, and training for motor function, and all 3 training courses were utilized in this study. However, the items in training for motor function that include movements directly applied to daily activities were excluded. In addition, “Joystim Man Special Agent Mission” in training for daily activities was also excluded because it had minimal relation to actual daily living. The difficulty level of the program was classified into stages 1 to 5, and the difficulty level was applied to each subject according to the subject’s physical function and cognitive function level.

##### 2.3.1.1. Activities of daily living training course.

Activities of daily living (ADL) training course consists of making cookies, making pizza, bathing, face washing, and hair washing. The correct order of the task, selection of necessary equipment for the task, and method of task performance can be trained.

##### 2.3.1.2. Cognition training course (cognition course).

The cognition training course consists of visual memory, counting the number of pictures, naming pictures, placing pictures in the correct order, basic arithmetic operations, completing pictures, finding words, reading the time on the clock, disc piles, serial number squares, finding missing objects, and pairing pictures. Visual perception, concentration, memory, eye-hand coordination, recognition, and language, which are part of the cognitive function, can be trained.

##### 2.3.1.3. Motor training course (MOTO course).

The motor training course consists of pressing piano keys, turning a jam jar, opening doors with round doorknobs, opening doors with “L” shaped handles, opening doors with automatic door locks, and nailing. Using various equipment, gripping power, multiple types of Grip and Pinch, forearm rotation and finger movements can be trained.

### 2.4. Measures

#### 2.4.1. Assessment of motor and process skills (AMPS).

AMPS is an assessment tool designed by Fisher (2006) and is used to assess the performance quality of daily activity tasks through task performance. Out of 116 standardized tasks, 2 to 3 significant tasks that are actually being performed by the subject are selected, and these are assessed in 4-point scales for motor skills (16 items) and process skills (20 items).^[38]^ For each skill item, raw score is entered into OTAP software, and this will be converted into an interval scale of logit. In this study, AMPS is utilized to measure the changes in the performance level of daily activities of subjects. Out of 116 AMPS standardized tasks, a total of 8 tasks including 4 easy tasks and 4 difficult tasks were selected through an interview between the investigator and the subject. In order to prevent the learning effect, 1 easy task and 1 difficult task were randomly selected and assessed for each session.

#### 2.4.2. Computer cognitive screening assessment system (COSAS).

COSAS is a computer-based cognitive assessment computerized tool designed to self-test and check cognitive impairments in normal elderly people and patients with brain damage. It consists of 6 fields, orientation, memory, attention, visual perception, language, and higher perception, with a total of 29 questions. The points for each question are added up to the total score. The range of the score is 0 to 100, and higher scores signify better cognitive function. In this study, COSAS is utilized to measure the changes in the cognitive function of subjects.

#### 2.4.3. Box and block test (BBT).

BBT is a tool for measuring gross motor function and manual dexterity, and it is performed by transferring 1-inch-sized blocks from one box to another. The score is equal to the number of blocks each hand had transferred for 1 minute.^[1]^ In this study, BBT is utilized to measure the changes in the upper extremities function of subjects.

#### 2.4.4. Grip and pinch strength test (GPST).

GPST measures 4 items (power grip, lateral pinch, tip pinch, 3-jaw pinch) in kilograms. A hand dynamometer and pinch gauge were used as measuring equipment, and measurements were taken with the shoulder adducted and the elbow flexed at 90°, with the lower arm in a neutral position.^[1]^ In this study, GPST is utilized to measure the changes in the upper extremities function of subjects. In all assessments, the subjects were instructed to grip the dynamometer as hard as possible, and the average value of 3 measurements taken at 1-minute intervals was used.

### 2.5. Statistical analysis

A visual graph is provided for the repetitive measurement results of daily activity performance, cognitive function, and upper extremities function for each session. Two standard deviation bands and trend line were used as the analysis method for significance testing. In order to interpret and compare the inclination of each visual graph objectively, terms such as “effect maximization,” “elevation,” “maintenance,” “elevation after decrease,” “gradual decrease,” and “limited effect” were used to compare and analyze the inclination of graphs for each session.

## 3. Results

### 3.1. Performance level of ADL for Intervention 1

Subject 1 showed a decrease in motor skills and an elevation in process skills in the performance of ADL for Intervention 1 during the period of Baseline A. Both motor and process skills showed elevation during Intervention period B, and 2 consecutive measurements were higher than the reference line of the 2 standard deviation bands from the Baseline A period. Both motor and process skills were higher in Second Baseline A’ period compared to the Baseline A period, and lower compared to Intervention period B. Both motor and process skills were higher in the Second Intervention period B’ compared to Second Baseline A’ period, with 2 consecutive measurements being higher than the reference line of the 2 standard deviation band, and the inclination slope was more gradual compared to Intervention period B.

**Figure 2.**
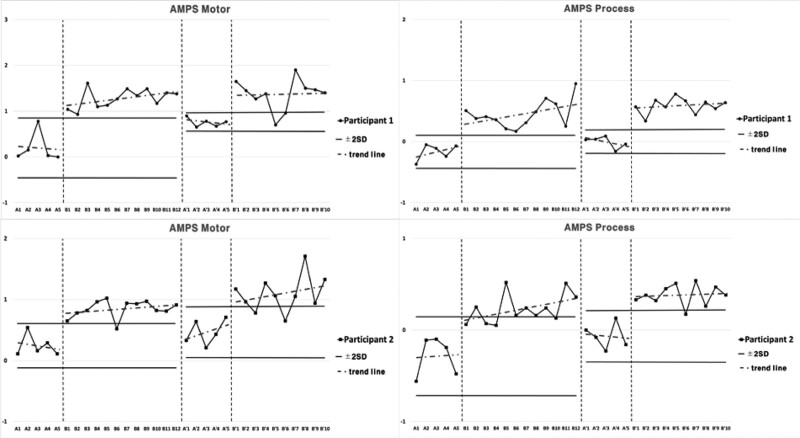
Results of ADL for Intervention 1. ADL = activities of daily living.

Subject 2 showed a decrease in motor skills and an elevation in process skills during Baseline A period. Both motor and process skills showed elevation during Intervention period B, and 2 consecutive measurements were higher than the reference line of the 2 standard deviation bands from the Baseline A period. Both motor and process skills were higher in Second Baseline A’ period compared to Baseline A period, and lower compared to Intervention period B. Both motor and process skills were higher in the Second Intervention period B’ compared to Second Baseline A’ period, 2 consecutive measurements being higher than the reference line of the 2 standard deviation bands. The inclination for motor skills was steeper compared to Intervention period B, and the inclination for process skills was more gradual compared to Intervention period B (Fig. 2; Table 2).

**Table 2:** Average value of assessment of motor and process skills for each session in Intervention 1.

		A	B	A’	B’
Participant 1	Motor skills	0.20	1.28	0.75	1.37
	Process skills	−0.17	0.45	−0.01	0.59
Participant 2	Motor skills	0.24	0.84	0.46	1.09
	Process skills	−0.29	0.23	−0.07	0.38

### 3.2. Performance level of ADL for Intervention 2

Subject 3 showed elevated inclination in both motor skills and process skills in the performance of ADL for Intervention 2 during the period of Baseline A. Both motor and process skills showed elevation during Intervention period B, and 2 consecutive measurements were higher than the reference line of the 2 standard deviation bands from Baseline A period. Both motor and process skills were higher in Second Baseline A’ period compared to Baseline A period, and lower compared to Intervention period B. Both motor and process skills were higher in Second Intervention period B’ compared to Second Baseline A’ period, 2 consecutive measurements being higher than the reference line of the 2 standard deviation bands. The inclination was more gradual compared to Intervention Period B.

**Figure 3.**
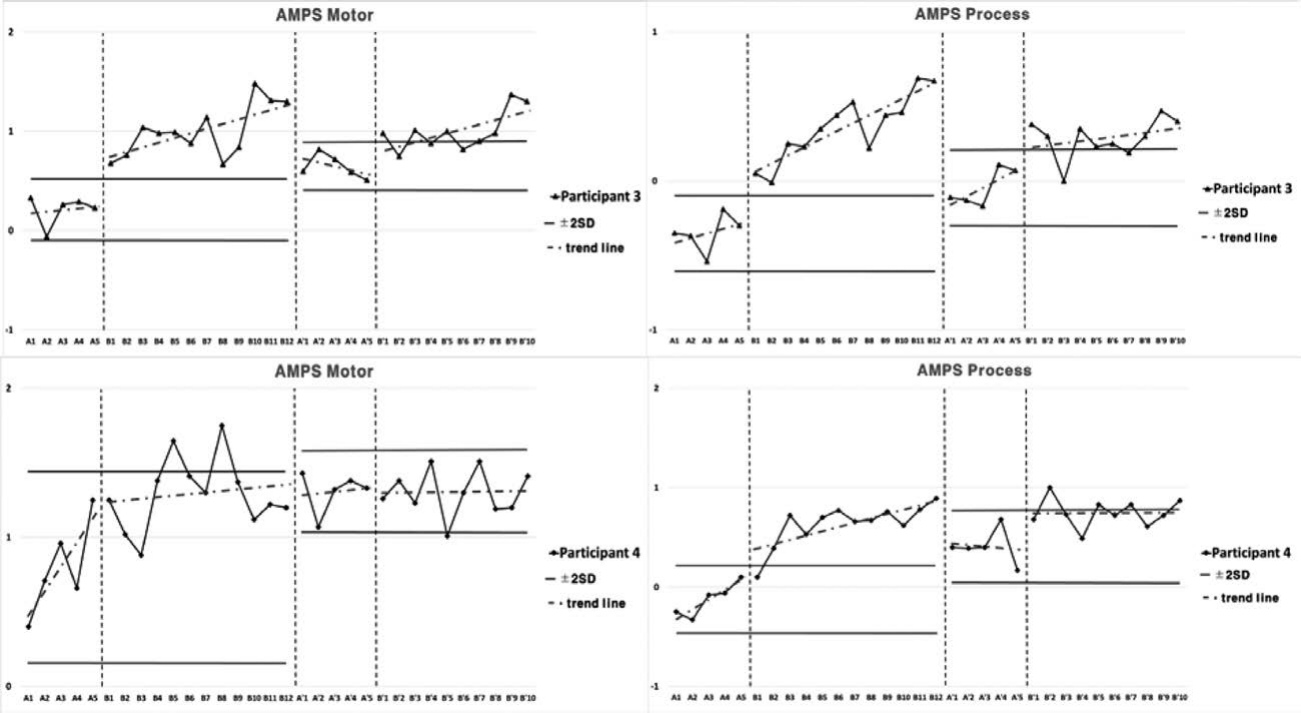
Results of ADL for Intervention 2. ADL = activities of daily living.

Subject 4 showed elevated inclination in both motor and process skills during Baseline A period. As for motor skills, Baseline A was observed to be unstable. Both motor and process skills showed elevated inclination during Intervention period B. For process skills 2 consecutive measurements were maintained higher than the reference line of the 2 standard deviation bands from Baseline A period, but higher measurements were not maintained for motor skills. Both motor and process skills were higher in Second Baseline A’ period compared to Baseline A period, and lower compared to Intervention period B. Two consecutive measurements of both motor and process skills were not maintained higher in Second Intervention period B’ compared to the reference line of 2 standard deviation bands from Second Baseline A’ period, and the inclination was more gradual compared to Intervention period B (Fig. 3; Table 3).

**Table 3:** Average value of assessment of motor and process skills for each session in Intervention 2.

		A	B	A’	B’
Participant 3	Motor skills	0.21	1.01	0.65	1
	Process skills	−0.35	0.36	−0.05	0.29
Participant 4	Motor skills	0.80	1.30	1.31	1.30
	Process skills	−0.12	0.63	0.41	0.75

### 3.3. Cognitive function and upper extremities function for Intervention 1

Subject 1 showed decreased inclination in cognitive function for Intervention 1 during the period of Baseline A. The inclination was elevated in Intervention period B, and 2 consecutive measurements were higher than the reference line of the 2 standard deviation bands from Baseline A period. For the Second Baseline A’ period, the results were higher than Baseline A period and lower than Intervention period B. As for the results of Second Intervention period B’, 2 consecutive measurements were not maintained higher than the reference line of 2 standard deviation bands from Second Baseline A’ period, and the inclination was steeper compared to Intervention period B.

Subject 1 showed elevated inclination in manual dexterity for Intervention 1 during the period of Baseline A. The inclination was elevated in Intervention period B, and 2 consecutive measurements were higher than the reference line of the 2 standard deviation bands from Baseline A period. For the Second Baseline A’ period, the results were higher than Baseline A period and lower than Intervention period B. The results were higher in Second Intervention period B’ compared to Second Baseline A’ period, 2 consecutive measurements being higher than the reference line of the 2 standard deviation bands, and the inclination was more gradual compared to Intervention period B.

Subject 1 showed constant inclination in power grip and elevated inclination in lateral pinch, tip pinch, and 3-jaw pinch in upper extremities strength for Intervention 1 during the period of Baseline A. All power grip, lateral pinch, tip pinch, and 3-jaw pinch showed elevated inclination in Intervention period B, and 2 consecutive measurements were higher than the reference line of 2 standard deviation bands from Baseline A period. For the Second Baseline A’ period, the results were higher than Baseline A period and lower than Intervention period B. The results were higher in Second Intervention period B’ compared to Second Baseline A’ period, with 2 consecutive measurements being higher than the reference line of the 2 standard deviation bands. The inclination was more gradual for power grip and tip pinch compared to Intervention period B, and steeper for lateral pinch and 3-jaw pinch.

Subject 2 showed elevated inclination in cognitive function for Intervention 1 during the period of Baseline A. The inclination was elevated in Intervention period B, and 2 consecutive measurements were higher than the reference line of the 2 standard deviation bands from Baseline A period. For the Second Baseline A’ period, the results were higher than Baseline A period and lower than Intervention period B. The results were higher in Second Intervention period B’ compared to Second Baseline A’ period, 2 consecutive measurements being higher than the reference line of the 2 standard deviation bands, and the inclination was more gradual compared to Intervention period B.

Subject 2 showed decreased inclination in manual dexterity for Intervention 1 during the period of Baseline A. The inclination was elevated in Intervention period B, and 2 consecutive measurements were higher than the reference line of the 2 standard deviation bands from Baseline A period. For the Second Baseline A’ period, the results were higher than Baseline A period and lower than Intervention period B. The results were higher in Second Intervention period B’ compared to Second Baseline A’ period, 2 consecutive measurements being higher than the reference line of the 2 standard deviation bands, and the inclination was more gradual compared to Intervention period B.

**Figure 4.**
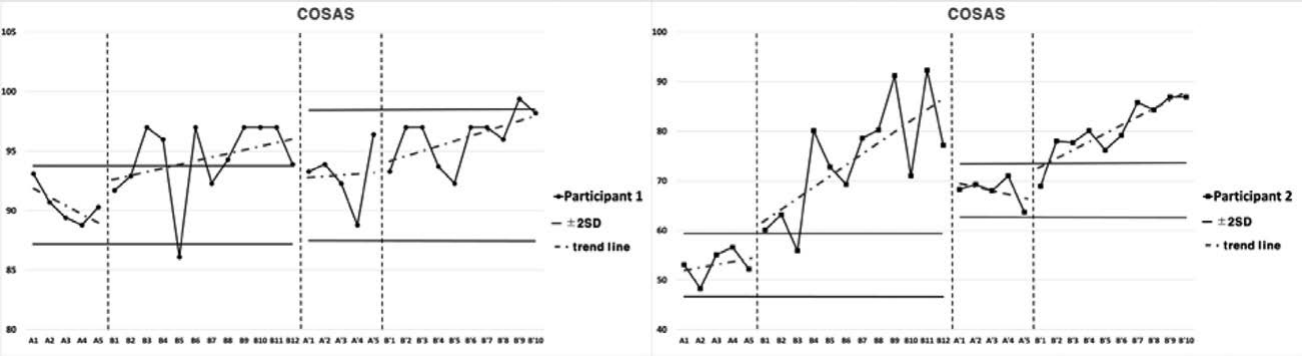
Results of cognitive functions for Intervention 1.

**Figure 5.**
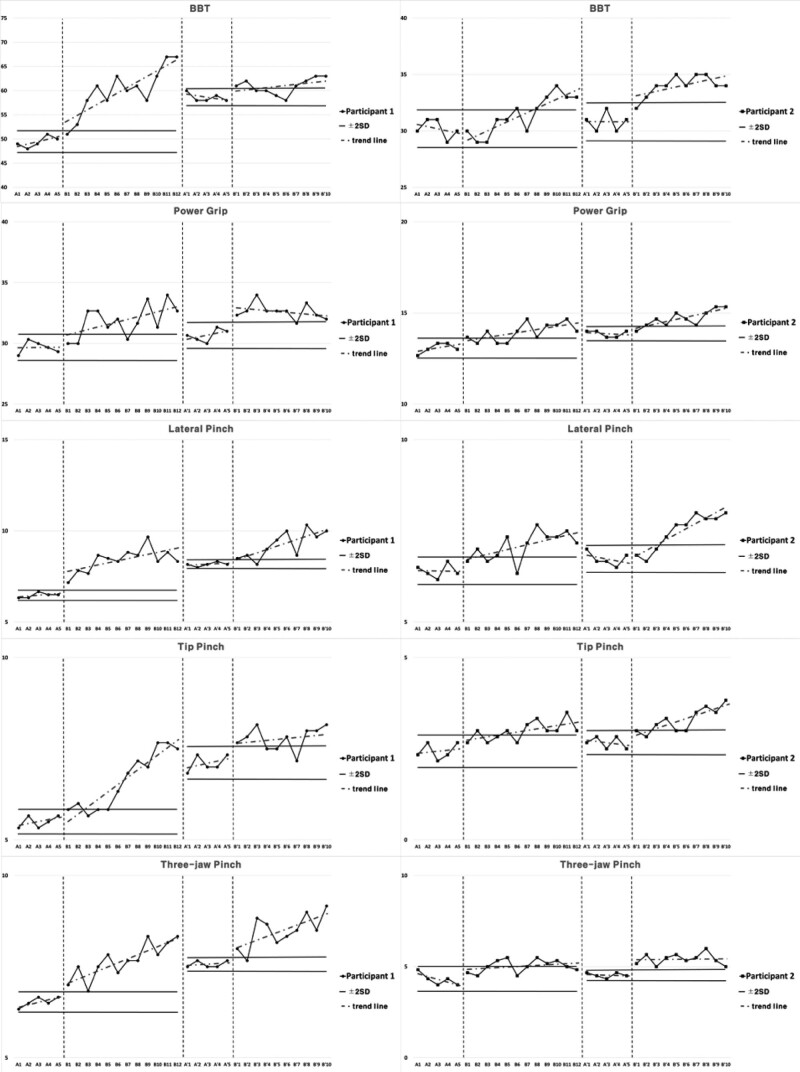
Results of hand functions for Intervention 1.

Subject 2 showed constant inclination in lateral pinch, elevated inclination in power grip and tip pinch, and decreased inclination in 3-jaw pinch in the upper extremities strength for Intervention 1 during the period of Baseline A. The inclination was elevated for power grip, lateral pinch, tip pinch, and 3-jaw pinch in Intervention period B, and 2 consecutive measurements were higher than the reference line of 2 standard deviation bands from Baseline A period. For the Second Baseline A’ period, the results were higher than Baseline A period and lower than Intervention period B. The results were higher in Second Intervention period B’ compared to Second Baseline A’ period, 2 consecutive measurements being higher than the reference line of the 2 standard deviation bands. The inclination was steeper for power grip, lateral pinch, and tip pinch compared to Intervention period B and more gradual for 3-jaw pinch. Cognitive and upper extremities function for intervention 1 are presented in Figure 4, Figure 5, and Table 4.

**Table 4:** Average value of cognitive and upper extremity functions for each session in Intervention 1.

			A	B	A’	B’
Cognitive functions	Participant 1	COSAS	90.46	94.35	92.94	96.09
	Participant 2	COSAS	53.06	74.33	68.06	80.4
Upper extremities functions	Participant 1	BBT	49.4	60	58.6	60.9
	Participant 2	BBT	30.2	31.41	30.8	34
	Participant 1	Power grip	29.67	31.86	30.67	32.63
		Lateral pinch	6.47	8.4	8.17	9.25
		Tip pinch	5.5	6.61	7.1	7.78
		3-jaw pinch	6.53	7.67	7.57	8.48
	Participant 2	Power grip	13.07	13.94	13.87	14.7
		Lateral pinch	6.4	7.08	6.73	7.48
		Tip pinch	2.43	2.99	2.67	3.28
		3-jaw pinch	4.3	5.03	4.53	5.42

BBT = box and block test, COSAS = computer cognitive screening assessment system.

### 3.4. Cognitive function and upper extremities function for Intervention 2

Subject 3 showed elevated inclination in cognitive function for Intervention 2 during the period of Baseline A. The inclination was elevated in Intervention period B, and 2 consecutive measurements were higher than the reference line of 2 standard deviation bands from Baseline A period. For the Second Baseline A’ period, the results were higher than Baseline A period and lower than Intervention period B. The results were higher in Second Intervention period B’ compared to Second Baseline A’ period, 2 consecutive measurements being higher than the reference line of 2 standard deviation bands, and the inclination was more gradual compared to Intervention period B.

Subject 3 showed elevated inclination in manual dexterity for Intervention 2 during the period of Baseline A. The inclination was elevated in Intervention period B, and 2 consecutive measurements were higher than the reference line of 2 standard deviation bands from Baseline A period. For the Second Baseline A’ period, the results were higher than Baseline A period and lower than Intervention period B. The results were higher in Second Intervention period B’ compared to Second Baseline A’ period, 2 consecutive measurements being higher than the reference line of 2 standard deviation bands, and the inclination was steeper compared to Intervention period B.

Subject 3 showed constant inclination in tip pinch, and elevated inclination in power grip, lateral pinch, and 3-jaw pinch in upper extremities strength for Intervention 2 during the period of Baseline A. The inclination was elevated in Intervention period B for power grip, lateral pinch, tip pinch, and 3-jaw pinch, and 2 consecutive measurements were higher than the reference line of 2 standard deviation bands from Baseline A period. For the Second Baseline A’ period, the results were higher than Baseline A period and lower than Intervention period B. The results were higher in Second Intervention period B’ compared to Second Baseline A’ period, 2 consecutive measurements being higher than the reference line of 2 standard deviation bands, and the inclination was more gradual compared to Intervention period B for lateral pinch, tip pinch, and 3-jaw pinch, and steeper for power grip.

Subject 4 showed decreased inclination in cognitive function for Intervention 2 during the period of Baseline A. The inclination was constant in Intervention period B, and 2 consecutive measurements were not maintained higher than the reference line of 2 standard deviation bands from Baseline A period. For the Second Baseline A’ period, the results were of similar level to Baseline A period and Intervention period B. The results were higher in Second Intervention period B’ compared to Second Baseline A’ period, 2 consecutive measurements being higher than the reference line of 2 standard deviation bands, and the inclination was steeper compared to Intervention period B.

Subject 4 showed elevated inclination in manual dexterity for Intervention 2 during the period of Baseline A. The inclination was elevated in Intervention period B, and 2 consecutive measurements were higher than the reference line of 2 standard deviation bands from Baseline A period. For the Second Baseline A’ period, the results were higher than Baseline A period and lower than Intervention period B. The results were higher in Second Intervention period B’ compared to Second Baseline A’ period, 2 consecutive measurements being higher than the reference line of 2 standard deviation bands, and the inclination was steeper compared to Intervention period B.

**Figure 6.**
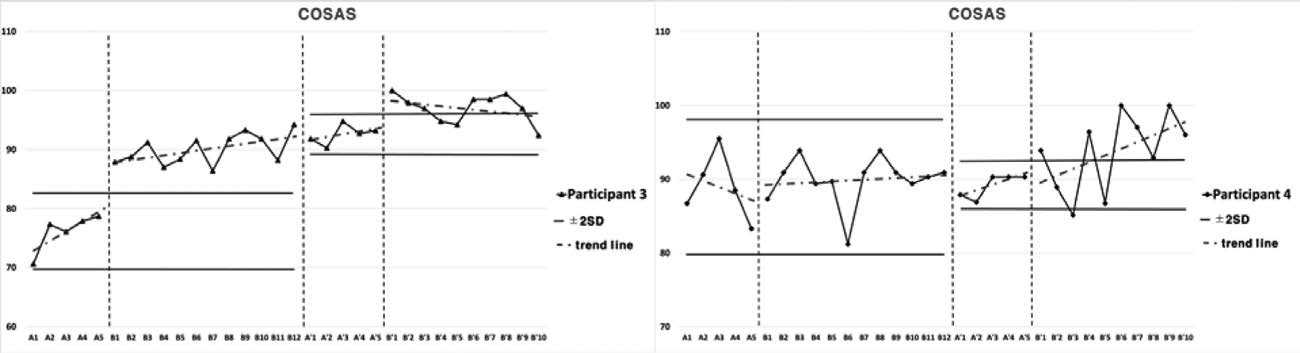
Results of cognitive functions for Intervention 2.

**Figure 7.**
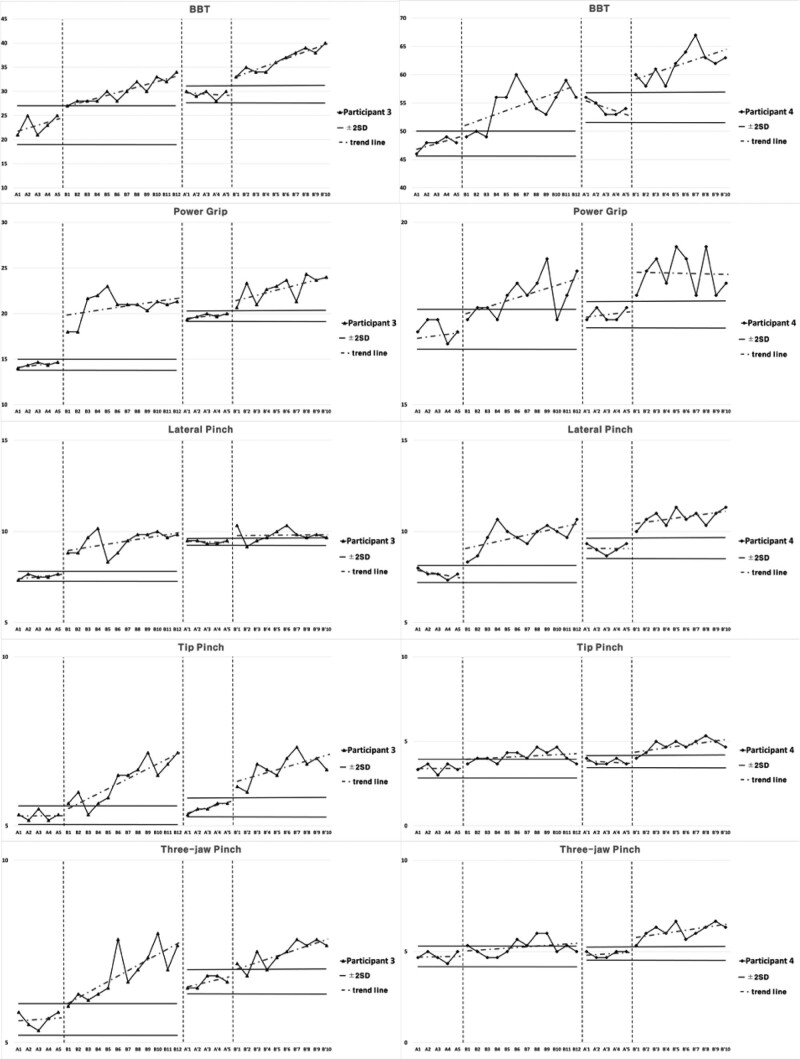
Results of hand functions for Intervention 2.

Subject 4 showed elevated inclination in the power grip, decreased inclination in lateral pinch, and constant inclination in tip pinch and 3-jaw pinch in upper extremities strength for Intervention 2 during the period of Baseline A. The inclination was elevated in Intervention period B for power grip, lateral pinch, tip pinch, and 3-jaw pinch. Two consecutive measurements were higher than the reference line of 2 standard deviation bands from Baseline A period. For the Second Baseline A’ period, the results were higher than Baseline A period and lower than Intervention period B. The results were higher in Second Intervention period B’ compared to Second Baseline A’ period, 2 consecutive measurements being higher than the reference line of 2 standard deviation bands, and the inclination was more gradual compared to Intervention period B for power grip and lateral pinch, and steeper for tip pinch and 3-jaw pinch. Cognitive and upper extremities function for intervention 2 are presented in Figure 6, Figure 7, and Table 5.

**Table 5:** Average value of cognitive and upper extremity functions for each session in Intervention 2.

			A	B	A’	B’
Cognitive	Participant 3	COSAS	76.12	90.04	92.56	96.97
functions	Participant 4	COSAS	88.92	89.89	89.14	93.69
Upper extremities functions	Participant 3	BBT	23	30	29.4	36.4
	Participant 4	BBT	47.8	54.58	54.2	61.8
	Participant 3	Power grip	14.4	20.81	19.73	22.77
		Lateral pinch	7.53	9.44	9.43	9.8
		Tip pinch	5.3	6.32	5.53	6.7
		3-jaw pinch	5.63	6.9	6.67	7.43
	Participant 4	Power grip	17.07	17.97	17.47	18.60
		Lateral pinch	7.67	9.75	9.07	10.77
		Tip pinch	3.4	4.11	3.8	4.77
		3-jaw pinch	4.73	5.25	4.87	6.13

BBT = box and block test, COSAS = computer cognitive screening assessment system.

### 3.5. Comparing inclinations of changes in performance of ADL, cognitive function, and upper extremities function for Interventions 1 and 2

Table 5 presents a comprehensive visual analysis comparing the inclination of graphs showing changes in the performance of ADL, cognitive function, and upper extremities function at each session following the application of Interventions 1 and 2. The performance of ADL following Intervention 1 showed 1 change from elevation to effect maximization, and 3 changes from elevation to maintenance. Following Intervention 2, 1 change from limited effect to limited effect, 1 change from elevation to limited effect, and 2 changes from elevation to elevation after decrease were confirmed. The effect of Intervention 1 on cognitive function and upper extremities function was confirmed as 1 change from elevation to limited effect, 1 change from elevation to gradual decrease, 2 changes from elevation to elevation after decrease, 3 changes from elevation to maintenance, and 5 changes from elevation to maintenance. The effect of Intervention 2 was confirmed to be 1 change from limited effect to elevation, 2 changes from elevation to gradual decrease, 2 changes from elevation to elevation after decrease, 2 changes from elevation to maintenance, and 5 changes from elevation to effect maximization (Table 6).

**Table 6:** Results of graph analysis for each intervention.

Intervention 1				Intervention 2			
Motor skills of ADL	1	Elevation→maintenance	Elevation→Effect maximization(1)	Motor skills of ADL	3	Elevation→Elevation after decrease	Limited effect→Limited effect (1)
	2	Elevation→ Effect maximization	Elevation→Maintenance (3)		4	Limited effect→ Limited effect	Elevation→Limited effect (1) Elevation→Elevation
Process skills of ADL	1	Elevation→ Maintenance		Process skills of ADL	3	Elevation→ Elevation after decrease	after decrease (2)
	2	Elevation→ Maintenance			4	Elevation→ Limited effect	
COSAS	1	Elevation→ Limited effect	Elevation→ Limited effect (1)	COSAS	3	Elevation→ Gradual decrease	Limited effect→ Elevation (1)
	2	Elevation→ Elevation after decrease	Elevation→ Gradual decrease (1)		4	Limited effect→ Elevation	Elevation→ Gradual decrease (2)
BBT	1	Elevation→ Elevation after decrease	Elevation→ Elevation after decrease (2)	BBT	3	Elevation→ Effect maximization	Elevation→ Elevation after decrease (2)
	2	Elevation→ Maintenance	Elevation→ Maintenance (3)		4	Elevation→ Effect maximization	Elevation→ Maintenance (2)
Power grip	1	Elevation→ Gradual decrease	Elevation→ Effect maximization (5)	Power grip	3	Elevation→ Effect maximization	Elevation→ Effect maximization (5)
	2	Elevation→ Effect maximization			4	Elevation→ Gradual decrease	
Lateral pinch	1	Elevation→ Effect maximization		Lateral pinch	3	Elevation→ Maintenance	
	2	Elevation→ Effect maximization			4	Elevation→ Maintenance	
Tip pinch	1	Elevation→ Maintenance		Tip pinch	3	Elevation→ Elevation after decrease	
	2	Elevation→ Effect maximization			4	Elevation→ Effect maximization	
3-jaw pinch	1	Elevation→ Effect maximization		3-jaw pinch	3	Elevation→ Elevation after decrease	
	2	Elevation→ Maintenance			4	Elevation→ Effect maximization	

(N) = the number of observations for each inclination change, ADL = activities of daily living, BBT = box and block test, COSAS = computer cognitive screening assessment system.

## 4. Discussion

This study was conducted to confirm whether VR-based intervention is effective for the improvement of daily activity performance, cognitive function, and upper extremities function. It also sought to compare and analyze the difference between VR-based daily activities training and cognitive and motor training for enhancing independence in stroke patients. The subjects of this study were 4 stroke patients, who have been diagnosed at least 6 months prior to the study. In order to compare the VR-based daily activities training with cognitive and motor training for enhancing independency in patients, the subjects were divided into 2 groups with 2 subjects each, and 2 different intervention methods were applied. AMPS, COSAS, BBT, and GPST were performed to measure the changes in the performance of daily activities, cognitive function, and upper extremities function.

The results of this study confirmed that VR-based intervention is effective in improving daily activity performance, cognitive function, and upper extremities function in stroke patients. For Subjects 1 and 2, to whom Intervention 1 has been applied, only the daily activities training was applied during Intervention period B and both the daily activities training and cognitive and motor function training were applied during the Second Intervention period B’. Subject 1 showed improvement in both motor and process skills in Intervention period B, and this was maintained in Second Intervention period B’. Cognitive function improved during Intervention period B, but the effect was inadequate during Second Intervention period B’. Manual dexterity improved during Intervention period B and improved after decrease during Second Intervention period B’. Upper extremities function was improved during Intervention period B, and during Second Intervention period B’, power grip showed a gradual decrease, tip pinch was maintained for the performance of daily activities, and lateral pinch and 3-jaw pinch were maximized. Subject 2 showed improvement in motor skills during Intervention period B and maximization of effect during Second Intervention period B’ for the performance of daily activities. Process skills showed improvement during Intervention period B and were maintained during Second Intervention period B’. Cognitive function improved during Intervention period B, and showed a tendency to improve after decrease during Second Intervention period B’. Manual dexterity improved during Intervention period B and was maintained during Second Intervention period B’. Upper extremities function improved during Intervention period B, and the effect was maintained during Second Intervention period B’ for 3-jaw pinch, and maximized for power grip, lateral pinch, and tip pinch.

For Subjects 3 and 4, to whom Intervention 2 has been applied, only the cognitive and motor function training was applied during Intervention period B and both the daily activities training and cognitive and motor function training were applied during the Second Intervention period B’. Subject 3 showed improvement in both motor and process skills for the performance of daily activities during Intervention period B, and improvement after a decrease during Second Intervention period B’. Cognitive function improved during Intervention period B, and showed a gradual decrease during Second Intervention period B’. Manual dexterity improved during Intervention period B, and the effect was maximized during Second Intervention period B’. Upper extremities function improved during Intervention period B, and during Second Intervention period B’, the effect was improved after decrease for tip pinch and 3-jaw pinch, maintained for lateral pinch, and maximized for power grip. Subject 4 showed an inadequate effect in motor skills for the performance of daily activities during Intervention period B, and the inadequacy was maintained during Second Intervention period B’. Process skills improved during Intervention period B, but the effect was inadequate during Second Intervention period B’. Cognitive function showed inadequate effect during Intervention period B, but there was a tendency for improvement during Second Intervention period B’. Manual dexterity was improved during Intervention period B, and the effect was maximized during Second Intervention period B’. Upper extremities function improved during Intervention period B, and during Second Intervention period B’, the effect gradually decreased for power grip, was maintained for lateral pinch, and maximized for tip pinch and 3-jaw pinch.

Although each subject showed varying results, no subject showed a decrease in function due to VR-based intervention, so it is considered to have a positive influence on the improvement of daily activities performance, cognitive function, and upper extremities function in stroke patients. However, in the case of AMPS motor for subject 4, the effects of the intervention were difficult to confirm because it was conducted in an unstable state during Baseline A, in order to take the measurements under uniform conditions according to the study design. The effect on COSAS of subject 1 and AMPS process of subject 4 was not confirmed because 2 consecutive measurements during Second Intervention period B’ were not higher than the reference line of 2 standard deviation bands from Second Baseline A’. In addition, 2 consecutive measurements for COSAS of subject 4 during Intervention period B did not remain higher than the reference line of 2 standard deviation bands from Baseline A. The subjects showed differences in the result, and especially in the case of subject 4, the age was comparatively younger than other subjects. This age factor is considered to have influenced the instability of the baseline and the effect of the intervention.

Previous studies reported that VR-based intervention induced a significant improvement in the performance of ADL, corresponding to the results of this study, which showed that VR-based intervention is effective in improving the performance of daily activities.^[34,39,40]^ According to a prior study while the application of VR-based intervention using haptic force feedback improved the performance of daily activities in chronic stage stroke patients, some patients showed deterioration in the performance of daily activities due to the use of upper extremities on the damaged side.^[41]^ Other studies have reported that VR-based intervention was effective in improving cognitive and upper extremities function in stroke patients. According to a study that was conducted to investigate the effect of Joystim-type VR-based intervention on the cognitive function and performance of daily activities in stroke patients, the intervention group showed significantly larger improvement compared to the control group in attention and visual occupational memory, and in self-management for the performance of daily activities. This shows that Joystim-type VR-based intervention can have more of a positive effect on the improvement of cognitive function and performance of daily activities in stroke patients compared to general therapy.^[20]^

According to a study that investigated the effect of VR-based upper extremities training combined with real-time feedback on upper extremities function, the performance of daily activities, and position control, there was a statistically significant difference in manual muscular strength for power grip and lateral pinch, and a significant difference in Jebson-Taylor hand function test and Box and Block Test between the 2 groups.^[42]^ As for the strength of the upper extremities, while some studies cited that the effect was confirmed following the application of VR-based intervention,^[43,44]^ the other study reported that the strength of the upper extremities did not show a significant improvement, or that there was no consistency.^[45]^ The results from this study on VR-based motor training showed improvement in upper extremities strength contrary to the previous study. However, power grip showed inconsistent results, in accordance with the previous study.

Although many previous studies reported favorable results following the application of VR-based intervention to stroke patients, the evidence is not yet sufficient to support superiority compared to general therapy or the generalization of improvement in VR-based intervention to real-life environments. This study did not confirm superior results in generalization, but the effects of the intervention were confirmed to be maximized by the application of VR-based intervention utilizing tasks concerning ADL.

In addition, the results of this study confirmed that after analyzing the effect of VR-based training for ADL compared to cognitive function and motor training on enhancing the independence of stroke patients, an early approach to training for ADL maximized the effect of the intervention. Comparing the effects of Intervention 1 (training for ADL was applied first and functional training was added later) and Intervention 2 (functional training was applied first and training for ADL was applied later), the effect on AMPS motor independency was elevated in the early stages and maintained or maximized for Intervention 1. The effect was elevated in the early stages and decreased and elevated again later, or if the effect was inadequate in the early stages, the inadequacy was maintained for Intervention 2. As for AMPS process independence, if the effect was elevated in the early stages, this was maintained for Intervention 1. For Intervention 2, if the effect was elevated in the early stages, it was inadequate or decreased and elevated again later. If the effect on cognitive function was elevated in the early stages, it was inadequate or decreased and elevated again later for Intervention 1. However, if the effect was elevated in the early stages of Intervention 2, it was decreased gradually later, or if the effect was inadequate in the early stages, it was elevated later. If the effect on manual dexterity was elevated in the early stages, it decreased and became elevated again or maintained for Intervention 1. If the effect was elevated in the early stages of Intervention 2, the effect was maximized later. As for the upper extremities function, if the effect was elevated in the early stages of Intervention 1, it was gradually decreased, elevated after decrease, maintained, or maximized later. For Intervention 2, if the effect was elevated in the early stages, it was gradually decreased, elevated after decreasing, maintained, or maximized later.

The application of Intervention 1 resulted in 3 changes from elevation to maintenance and 1 change from elevation to maximization of effect for the performance of daily activities. However, for the cognitive and upper extremities function, there was 1 change from elevation to inadequate effect, 1 change from elevation to gradual decrease, 2 changes from elevation to elevation after decrease, 3 changes of maintenance after elevation, and 5 changes of maximization after elevation. Therefore, for Intervention 1, the effect on the performance of daily activities was elevated and maintained or maximized, but the effect on cognitive and upper extremities function was elevated and maintained, or in some cases, the effect was inadequate or decreased. The application of Intervention 2 resulted in 1 change from elevation to inadequate effect, 1 change of maintenance of inadequate effect, and 2 changes from elevation to elevation after decrease in performance of daily activities, however, for cognitive and upper extremities function, there were 2 changes from elevation to gradual decrease, 2 changes from decrease to elevation, 2 changes of maintenance, 5 changes of maximization, and 1 change from inadequate effect to elevation. Therefore, for Intervention 2, the effect on the performance of daily activities was elevated after decrease or inadequate. However, the effect on cognitive and upper extremities function was observed to be elevated and maximized in most cases, and inadequate or decreased in some cases.

The effect on the performance of daily activities was more maximized or elevated as result of Intervention 1 compared to Intervention 2, and the effect on cognitive and upper extremities function was more elevated and maintained compared to Intervention 2. Therefore, the early approach to daily activities training was confirmed to be the method that maximizes the effect of the intervention.

Task-oriented approach is a method of intervention that enhances the performance of daily activities by repetition of tasks necessary for daily living.^[46]^ It consists of tasks that can help improve the actual performance of daily activities when applied to stroke patients, providing a more efficient treatment option, and positively influencing the performance of daily activities by improving the performance of the targeted task using the nondamaged side along with the damaged side, rather than overcoming the issues of the damaged side.^[46,47]^ Reports from previous studies have confirmed improvement of function by application of task-oriented intervention through ADL. Task-oriented activities centered on training for ADL were more effective for the performance of daily activities and cognitive function in stroke patients compared to general rehabilitation training.^[48]^ And, task-oriented activities were more helpful in improving daily activities performance, balancing, and satisfactory task performance in chronic stroke patients compared to general occupational therapy.^[49]^ In addition, task-oriented approach can greatly influence the upper extremities function, and while each approach has its benefits, reinforcing the diversity of tasks by utilizing various stimuli, patient-centered goals, application of environments according to circumstances, and feedback is expected to have a higher level of therapeutic efficiency in functional improvement of patients, compared to uniform, typical tasks.^[50]^ The prior study investigated the effect of VR-based training focused on ADL on the upper extremities function and the performance of daily activities in stroke patients and reported that VR-based training focused on ADL was more beneficial for the upper extremities function and performance of daily activities in stroke patients compared to general VR-based training.^[37]^ This supports the theory of this study that early approach to training for ADL is more efficient. There are only a few studies that compare training for ADL and functional training, and investigate which intervention has a more significant effect on the performance of daily activities, cognitive function, and upper extremities function in stroke patients. Therefore, the significance of this study is that it provided the basis for adequate therapy to enhance independence and functional improvement in stroke patients.

The subjects who participated in this study showed improvement in daily activities performance, cognitive function, and upper extremities function after the application of VR-based intervention. Therefore, a VR-based rehabilitation program using MOTOCOG is considered to be a beneficial intervention for enhancing daily activities performance, cognitive function, and upper extremities function in stroke patients. The methods of intervention were designed differently in order to compare and analyze the effect of daily activities training and functional training. It was also confirmed that early approach to daily activities training was the more effective method of intervention.

This study has several limitations. First, it was a single-subject study with 4 participating subjects, so it is difficult to generalize the study results. Second, there was no consideration for the subject’s age and gender. The gender of all subjects was male, and no significant difference was confirmed in the intervention effect of 1 elderly and 1 young person. However, there is a limit to generalizing the results of failing to control demographic variables. Third, it is also difficult to generalize differences according to the diagnosis of stroke patients. Actually, no difference in the intervention effect was confirmed in patients with infarction including only 1 patient. In particular, this patient is elderly but has reported meaningful effects. Forth, in comparing the 2 different methods of intervention, the effect of daily activities training on the performance of daily activities and the effect of functional training on cognitive function and motor function was not compared. These issues need to be remedied by selecting subjects with a more definite set of criteria and designing a study adequate for generalization, leading to the conduct of a study with more objective verifications.

## 5. Conclusions

This study aimed to confirm the effect of VR-based intervention on the performance of daily activities, cognitive function, and upper extremities function. The effects of VR-based training for ADL on training for cognitive and motor function on the enhancement of independence in stroke patients were compared and analyzed. The results of the study are as follows.

First, VR-based intervention showed a positive effect on the performance of daily activities, cognitive function, and upper extremities function in stroke patients. The confirmation of the effect on generalization for adapting to daily living in stroke patients through VR-based intervention using tasks of daily living was clinically significant in particular.

Second, when the VR-based training for daily activities and training for cognitive and motor function was compared and analyzed, the early approach to training for daily activities maximized the effect of the intervention for enhancement of independency in stroke patients.

Through these results, VR-based intervention of training for daily activities and functional training can be considered to benefit the improvement of the performance of daily activities, cognitive function, and upper extremities function in stroke patients. In addition, although functional training was also effective in enhancing independency and functional improvement in stroke patients, early approach to training for ADL based on tasks with objectives was deemed to be more effective.

## Acknowledgments

The authors would like to thank all participants for their support on the data acquisition.

## Author contributions

**Conceptualization:** Lan-Ju Lee, Seong-Youl Choi.

**Data curation:** Lan-Ju Lee, Seong-Youl Choi.

**Formal analysis:** Lan-Ju Lee, Seong-Youl Choi, Sang-Woo Han.

**Investigation:** Lan-Ju Lee, Seong-Youl Choi.

**Methodology:** Lan-Ju Lee, Seong-Youl Choi.

**Supervision:** Seong-Youl Choi.

**Writing – original draft:** Lan-Ju Lee.

**Writing – review & editing:** Lan-Ju Lee, Seong-Youl Choi, Hye-Sun Lee, Sang-Woo Han.

## References

[1] Radomski MV Trombly CA. Occupational Therapy for Physical Dysfunction. 7th ed. Philadelphia: Lippincott Williams & Wilkins; 2014.

[2] Lawrence ES Coshall C Dundas R. Estimates of the prevalence of acute stroke impairments and disability in a multiethnic population. Stroke. 2001;32:1279–84. 11387487 10.1161/01.str.32.6.1279

[3] Wu CY Chen CL Tsai WC. A randomized controlled trial of modified constraint-induced movement therapy for elderly stroke survivors: changes in motor impairment, daily functioning, and quality of life. Arch Phys Med Rehabil. 2007;88:273–8. 17321816 10.1016/j.apmr.2006.11.021

[4] Kim YG. The effects of Korean computer-based cognitive rehabilitation program (CoTras) for the cognition and ADL in stroke. Korean J Occup Ther. 2011;19:75–88.

[5] Özdemir F Birtane M Tabatabaei R. Cognitive evaluation and functional outcome after stroke. Am J Phys Med Rehabil. 2001;80:410–5. 11399001 10.1097/00002060-200106000-00003

[6] Norlander A Jönsson AC Ståhl A. Activity among long-term stroke survivors. A study based on an ICF-oriented analysis of two established ADL and social activity instruments. Disabil Rehabil. 2016;38:2028–37. 26729231 10.3109/09638288.2015.1111437

[7] Wyller TB Holmen J Laake P. Correlates of subjective well-being in stroke patients. Stroke. 1998;29:363–7. 9472875 10.1161/01.str.29.2.363

[8] Kang DH. A comprehensive approach to occupational science. Korean J Occup Ther. 2000;8:121–7.

[9] Hsieh CL Hoffmann T Gustafsson L. The diverse constructs use of activities of daily living measures in stroke randomized controlled trials in the years 2005–2009. J Rehabil Med. 2012;44:720–6. 22772830 10.2340/16501977-1008

[10] Kang DH Kim JK Park SY. A correlation between the Canadian occupational performance measure and the assessment of motor and process skills: a pilot study. Korean J Occup Ther. 2005;13:25–35.

[11] Polatajko HJ McEwen SE Ryan JD. Pilot randomized controlled trial investigating cognitive strategy use to improve goal performance after stroke. Am J Occup Ther. 2012;66:104–9. 22389945 10.5014/ajot.2012.001784

[12] Kim KS Seo HM Kang JY. The effects of community based self-help management program on the activity of daily life, muscle strength, depression and life satisfaction of post-stroke patients. Korean J Rehabil Nurs. 2000;3:108–17.

[13] Roh KH. The effect of home rehabilitation exercise program of home stayed chronic hemiplegic stroke patients. J Korean Public Health Nurs. 2002;16:77–94.

[14] Cho MS Kim YH Yang YA. Basic survey for revitalize of employment of occupational therapists in community health center. Korean J Occup Ther. 2006;14:1–11.

[15] Yang YA Oh YH Hur JG. A study on policy of the home based occupational therapy for improvement of the old-age health. J Welf Aged. 2006;31:1598–649.

[16] Choi HS. Need on home-based occupational therapy of family caregivers with CVA Patients. Korean J Occup Ther. 2006;14:81–93.

[17] An TG Kim KU. Need on home-based occupational therapy and caregiving burden: busan and south gyeongsang areas centered around. Asia Cult Acad Inc Assoc. 2016;7:117–32.

[18] Jeong EH. Effect of home-visit occupational therapy on community dwelling stroke survivors: a case study. Ther Sci Rehabil. 2020;9:87–98.

[19] Bruin ED Schoene D Pichierri G. Use of virtual reality technique for the training of motor control in the elderly. Z Gerontol Geriatr. 2010;4:229–34. 10.1007/s00391-010-0124-7 20814798

[20] Yang NY Park HS Yoon TH. Effectiveness of motion-based virtual reality training (Joystim) on cognitive function and activities of daily living in patients with stroke. J Rehabil Welfare Eng Assist Technol. 2018;12:10–9.

[21] Rizzo AA Buckwalter JG Neumann U. Virtual reality and cognitive rehabilitation: a brief review of the future. J Head Trauma Rehabil. 1997;12:1–15.

[22] Cherniack EP. Not just fun and games: applications of virtual reality in the identification and rehabilitation of cognitive disorders of the elderly. Disabil Rehabil Assist Technol. 2011;6:283–9. 21158520 10.3109/17483107.2010.542570

[23] Camacho MLM. Virtual reality, a new tool for a new educational paradigm. Educ Media Int. 1998;35:226–71.

[24] Choi KB Cho SH. Convergence effect of virtual reality program on activities of daily living ability in stroke patients: meta-analysis. J Korea Converg Soc. 2020;11:63–70.

[25] Kim JH Oh MH Lee JS. The effects of training using virtual reality games on stroke patients’ functional recovery. Korean J Occup Ther. 2011;19:101–14.

[26] Crosbie JH Lennon S McGoldrick MC. Virtual reality in the rehabilitation of the arm after hemiplegic stroke: a randomized controlled pilot study. Clin Rehabil. 2012;26:798–806. 22275463 10.1177/0269215511434575

[27] Turolla A Dam M Ventura L. Virtual reality for the rehabilitation of the upper limb motor function after stroke: a prospective controlled trial. J Neuroeng Rehabil. 2013;10:851–9. 10.1186/1743-0003-10-85 PMC3734026 23914733

[28] Kim HG Lee MY Yang YA. Literature research on the clinical effect of the virtual reality-based rehabilitation program. J Occup Ther Aged Dement. 2018;12:1–11.

[29] Choi BG Kwon JS. A preliminary study of the effects of monitor-based virtual reality games on the cognition & activities of daily living for acute stroke: a double-blind randomized controlled trial. J Korea Contents Assoc. 2020;20:531–40.

[30] Won YS Chae SY Bak IH. The effect of motion-based virtual reality training on upper limb function in patients with acute stroke. J Rehabil Welf Eng Assist Technol. 2018;12:191–8.

[31] Lee MJ Koo HM. The effect of virtual reality-based sitting balance training program on ability of sitting balance and activities of daily living in hemiplegic patients. J Korean Soc Integr Med. 2017;5:11–9.

[32] Faria AL Andrade A Soares L. Benefits of virtual reality based cognitive rehabilitation through simulated activities of daily living: A randomized controlled trial with stroke patients. J Neuroeng Rehabil. 2016;13:1–12. 27806718 10.1186/s12984-016-0204-z PMC5094135

[33] Adams RJ Lichter MD Ellington A. Virtual activities of daily living for recovery of upper extremity motor function. IEEE Trans Neural Syst Rehabil Eng. 2017;26:252–60. 10.1109/TNSRE.2017.2771272 PMC12988643 29324411

[34] Kim YG. The effect of the virtual reality rehabilitation system on activities of daily living, cognitive function, self-esteem in stroke. J Korea Acad-Ind Cooperation Soc. 2015;16:5476–84.

[35] Kim SJ Chin ST Kim HT. Effects of whole-body vibration exercise and virtual reality training on balance and walking ability in the stroke patient. Korean J Growth Dev. 2014;22:151–7.

[36] Park YH Lee CH Lee BH. Clinical usefulness of the virtual reality-based postural control training on the gait ability in patients with stroke. J Exerc Rehabil. 2013;9:489–94. 24282810 10.12965/jer.130066 PMC3836554

[37] Park IH. Effect of virtual reality training focus on ADL on upper extremity function and activities of daily living in stroke patients. J Korea Entertainment Ind Assoc. 2019;13:321–9.

[38] Fisher AG Jones KB. Assessment of Motor and Process Skills: volume II-User Manual. Fort Collins, CO: Three Star Press; 2014.

[39] Laver K George S Thomas S. Cochrane review: virtual reality for stroke rehabilitation. Eur J Phys Rehabil Med. 2012;48:523–30. 22713539

[40] Merians AS Tunik E Fluet GG. Innovative approaches to the rehabilitation of upper extremity hemiparesis using virtual environments. Eur J Phys Rehabil Med. 2009;45:123–33. 19158659 PMC5648052

[41] Broeren J Rydmark M Björkdahl A. Assessment and training in a 3-dimensional virtual environment with haptics: a report on 5 cases of motor rehabilitation in the chronic stage after stroke. Neurorehabil Neural Repair. 2007;21:180–9. 17312093 10.1177/1545968306290774

[42] Jeon SY. The effect of virtual reality-based upper extremity rehabilitation combine with real time feedback training on upper extremity and postural control function in stroke patients (Master’s thesis). Seoul: Sahmyook University; 2017.

[43] Holden MK Dyar T. Virtual environment training-a new tool for neurorehabilitation? NR. 2002;26:62–71.

[44] Jang SH You SH Hallett M. Cortical reorganization and associated functional motor recovery after virtual reality in patients with chronic stroke: an experimenter-blind preliminary study. Arch Phys Med Rehabil. 2005;86:2218–23. 16271575 10.1016/j.apmr.2005.04.015

[45] Merians AS Poizner H Boian R. Sensorimotor training in a virtual reality environment: does it improve functional recovery poststroke? Neurorehabil Neural Repair. 2006;20:252–67. 16679503 10.1177/1545968306286914

[46] French B Leathley MJ Sutton CJ. A systematic review of repetitive task training with modelling of resource use, costs and effectiveness. Health Technol Assess. 2008;12:1–117. 10.3310/hta12300 18547501

[47] Almhdawi KA Mathiowetz VG White M. Efficacy of occupational therapy task-oriented approach in upper extremity post-stroke rehabilitation. Occup Ther Int. 2016;23:444–56. 27761966 10.1002/oti.1447

[48] Bang YS. The effects of task-oriented activities on the cognitive function and performance of activities of daily living in stroke patients. Korean J Occup Ther. 2007;15:49–61.

[49] Park SJ Bae SY. The effects of task-oriented activities on ADL performance, balance and satisfaction in chronic stroke patients. Korean J Occup Ther. 2012;20:1–11.

[50] Park SK Lee JS. Task-oriented approaches to improve upper limb functions and activities of daily living in stroke patients: systemic review and meta-analysis. Korean J Occup Ther. 2021;29:53–69.

